# Successful anesthesia management for middle cerebral artery thrombectomy in a patient with asynchronous cardio-cerebral infarction: a case report

**DOI:** 10.3389/fphar.2026.1711037

**Published:** 2026-02-24

**Authors:** Lin Zhang, Zhuang Liu, Tingting Wu, Shoushi Wang, Tingting Song

**Affiliations:** 1 Department of Anesthesia and Perioperative Medicine, Qingdao Central Hospital, University of Health and Rehabilitation Sciences (Qingdao Central Hospital), Qingdao, Shandong, China; 2 Operation Room, Qingdao Central Hospital, University of Health and Rehabilitation Sciences (Qingdao Central Hospital), Qingdao, Shandong, China; 3 Research Management Office, Qingdao Central Hospital, University of Health and Rehabilitation Sciences, Qingdao, China

**Keywords:** anesthesia management, asynchronous cardio-cerebral infarction, echocardiography, hemodynamic stability, middle cerebral artery thrombectomy

## Abstract

**Background:**

Cardio-cerebral infarction (CCI) is a rare syndrome characterized by acute ischemic stroke (AIS) occurring shortly after acute myocardial infarction (AMI). Currently, there are no evidence-based guidelines for perioperative anesthesia management in patients with CCI.

**Case summary:**

A 58-year-old male underwent coronary stent implantation for acute myocardial infarction (AMI) 4 days prior and was admitted for emergency mechanical thrombectomy due to occlusion of the right middle cerebral artery. Preoperative transthoracic echocardiography revealed left ventricular systolic dysfunction (ejection fraction <40%), segmental wall motion abnormalities, and a left ventricular apical thrombus, this case extends beyond simple post-myocardial infarction thromboembolism because the patient’s AIS occurred in the specific context of acute, severe cardiac dysfunction (EF <40%) with a documented left ventricular thrombus—a direct embolic source stemming from the recent AMI. This fulfills the criteria for “asynchronous cardio-cerebral infarction”, where the brain insult is a direct consequence of the cardiac event within a short temporal window. Anesthesia was managed using a non-intubated general anesthesia approach, involving titration of sedation with sufentanil and remifentanil, combined with norepinephrine to maintain mean arterial pressure (MAP) within ±20% of baseline. The risk of ischemia and hemorrhage was balanced with restrictive fluid management and continuous infusion of tirofiban. Intraprocedural hemodynamics remained stable, and the procedure was successfully completed. The patient was transferred to the general ward on postoperative day three and discharged on day eleven. Troponin I and brain natriuretic peptide (BNP) levels showed a downward trend, with no evidence of heart failure, hemorrhagic transformation, or acute kidney injury.

**Conclusion:**

In this case of CCI patients, immediate echocardiography was helpful in quickly assessing cardiac function and determining the source of the thrombus. Non-invasive general anesthesia was beneficial in maintaining hemodynamic stability and airway safety. The multidisciplinary individualized anesthesia plan developed in this challenging scenario may provide practical references for perioperative management of similar high-risk CCI patients, but its general applicability still needs to be verified in larger-scale studies.

## 1 Introduction

Cardio-cerebral infarction (CCI) is a rare clinical syndrome characterized by the successive or simultaneous occurrence of acute myocardial infarction (AMI) and acute ischemic stroke (AIS) within a short period. According to the temporal relationship of onset, CCI can be classified into two types: synchronous (AMI and AIS occur simultaneously) and asynchronous (AMI and AIS occur with an interval of several days to 3 months) (Ibekwe et al., 2022; Marto et al., 2019; Vaitkus and Barnathan, 1993; Aggarwal et al., 2021). Approximately 2.5% of patients with AMI experience stroke within 4 weeks of onset. Currently, clinical evidence for CCI mainly comes from case reports, and there is a lack of unified diagnostic criteria and treatment consensus. Here, we report a case of a CCI emergency patient who underwent coronary stent implantation due to “myocardial infarction” 4 days ago and is now scheduled for mechanical thrombectomy of the middle cerebral artery due to stroke. The successful implementation of middle cerebral artery thrombectomy in adult patients after myocardial infarction depends not only on the choice of anesthesia method but also on the assessment of perioperative cardiac function. The challenge for anesthesiologists is that they are required to perform surgery within minutes with almost no preoperative information, and anesthesia decisions usually need to be made rapidly within the first few minutes after the patient arrives at the operating room. Fifty percent of patients were aphasic, making it impossible to obtain their medical history, medication history, fasting time, and history of previous myocardial infarction. To address the issue of preoperative myocardial infarction in these patients, we should avoid significant fluctuations in preload, afterload, and heart rate as much as possible, and prevent heart failure. Unintubated general anesthesia causes smaller hemodynamic fluctuations during the induction period, and the non-intubation and extubation periods are more suitable for such patients than intubated general anesthesia. Perioperative transthoracic echocardiography is used to noninvasively and effectively assess the cardiac structure and function of the patient. Considering the high-risk nature of general anesthesia in such patients, we chose unintubated general anesthesia.

## 2 Case report

### 2.1 Case presentation

A 58-year-old male (height, 160 cm; weight, 68 kg) was scheduled for cerebral angiography and mechanical thrombectomy of the middle cerebral artery owing to occlusion of the right middle cerebral artery. Present illness: The patient was found to have slurred speech and inability to move the left limb 5 h prior and was transferred to our hospital for emergency treatment. The patient had a history of coronary stent implantation due to “myocardial infarction” 4 days ago. T, 36.2 °C; P, 80 beats/min; R, 17 breaths/min; BP, 120/75 mmHg; drowsiness, slight agitation, limited left eye movement, low muscle tone in the left limb, muscle strength 0, positive left Babinski sign, right limb muscle strength 5, negative Babinski sign, and negative meningeal irritation sign. Auxiliary examination: computed tomography angiography (CTA) of the brain showing occlusion of the right middle cerebral artery. Laboratory test results were unavailable.

**FIGURE 1:**
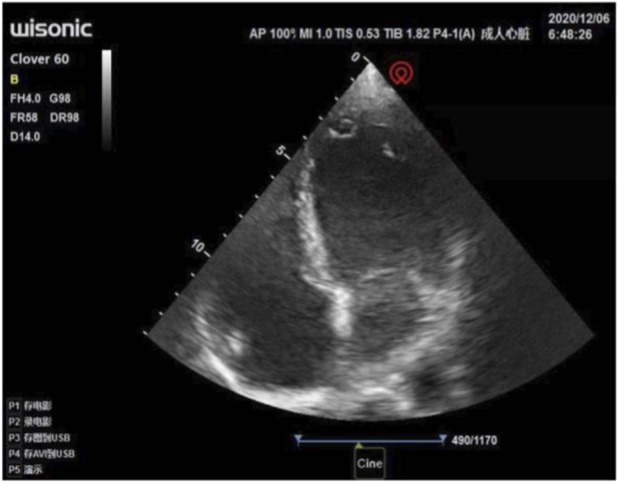
Transthoracic echocardiography shows segmental wall motion abnormalities, with decreased systolic activity in the anterior septum and the basal to apical segments of the anterior wall of the left ventricle.

**FIGURE 2:**
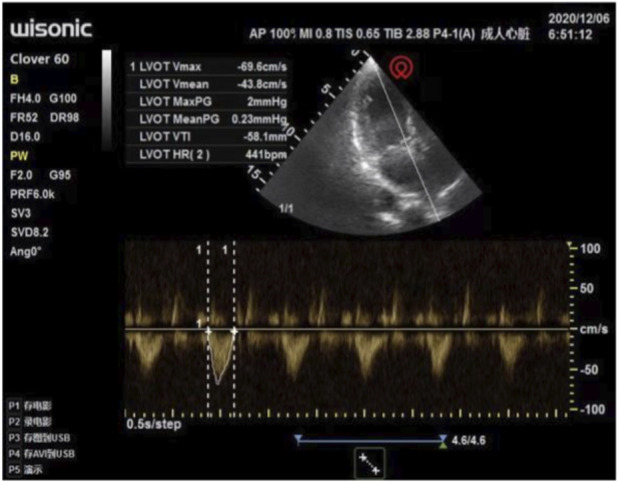
Reduced left ventricular systolic function: Left ventricular outflow tract VTI 11.6 cm, SV 36 mL, EPSS 1.43 cm, EF <40%.

**FIGURE 3:**
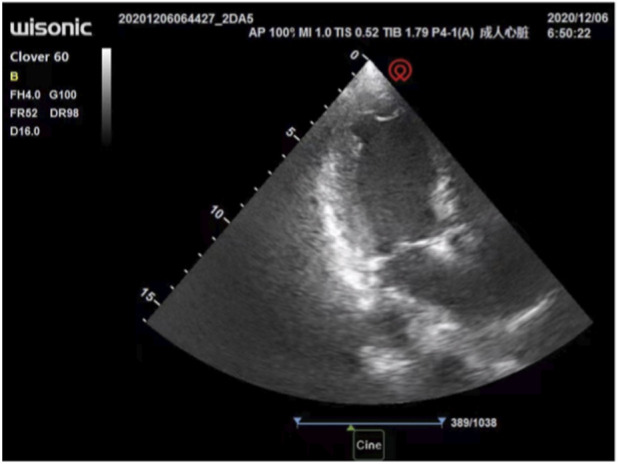
Attached substance at the apex of the left ventricle, suspected to be a thrombus.

**FIGURE 4:**
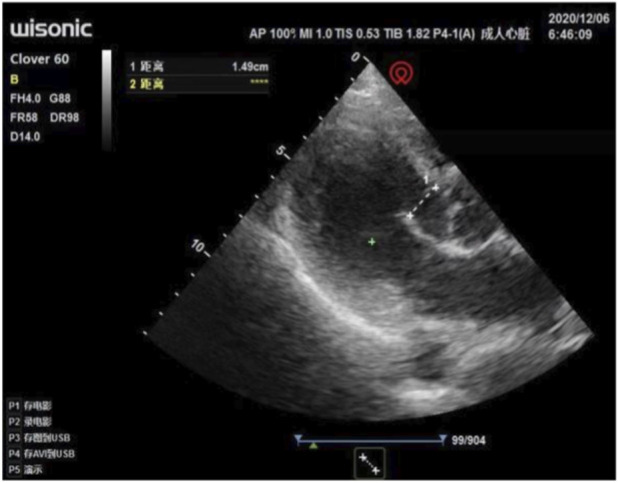
Mild regurgitation of the aortic valve and mitral valve.

The anesthesiologist used transthoracic echocardiography to assess cardiac structure and function before the operation. Standard transthoracic echocardiography was used to observe the heart from the apical view. Bedside ultrasound assessment results: ① segmental wall motion abnormalities, with decreased contraction activity in the anterior septum and the basal to apical segments of the anterior wall of the left ventricle (Figure 1); ② Decreased left ventricular systolic function: left ventricular outflow tract velocity-time integral (VTI) 11.6 cm, stroke volume (SV) 36 mL, E-point septal separation (EPSS) 1.43 cm, ejection fraction (EF) < 40% (Figure 2); ③ Left ventricular apical attachment, considered to be a thrombus (Figure 3); ④ Mild regurgitation of the aortic valve and mitral valve (Figure 4). Based on the medical history and ultrasound assessment results, we planned to administer unintubated anesthesia to the patient.

Before the operation, adrenaline, noradrenaline, milrinone, and isosorbide dinitrate vasoactive drugs were prepared, and routine monitoring was established, including noninvasive blood pressure, heart rate, blood oxygen saturation, and electrocardiogram. The patient’s vital signs were as follows: ventricular rate, 77 beats/min; invasive blood pressure, 112/78 mmHg; and respiratory rate, 18 breaths/min. The oxygen saturation in the breathing room air was 95%, and it was 98% when an oxygen mask was used. After entering the operating room, the patient was agitated. Sedation was induced with sufentanil 3 µg and maintained with a continuous infusion of remifentanil at 0.1 μg/kg/min. The patient fell asleep, and during the intraprocedural angiography, the right middle cerebral artery was occluded. The thrombus was removed using a thrombectomy stent, achieving successful reperfusion and thrombolysis in cerebral infarction (TICI) is grade 3. Intraprocedural vital signs were stable. During the operation, 400 mL lactated Ringer’s solution was injected. Tirofiban was continuously pumped into the intensive care unit (ICU) until admission. After the operation, the patient was conscious and was transferred to the ICU. On the day of admission to the ICU, the patient was conscious, communicating normally with cardiac enzymes: troponin I 7.2 ng/mL (critical value), BNP 932.78 pg/mL (critical value). A consultation from the cardiology department was requested, and tirofiban was continued. On the third postoperative day, the patient was conscious and fluent in speech without obvious discomfort. The left upper limb muscle strength was grade 0 and the left lower limb muscle strength was grade 1. The neurological recovery status of the patients was standardizedly evaluated using the National Institutes of Health Stroke Scale (NIHSS) and the Modified Rankin Scale (mRS). On the third day after the surgery, the NIHSS score was 8 points (mainly manifested as left-sided motor dysfunction), and the mRS score was 3 points, indicating that the patient had moderate disability and required some assistance but was still able to walk independently. The patient was transferred to the general ward. The six-day postoperative levels of cardiac troponin I (ng/mL) and BNP (pg/mL) were continuously detected (Table 1). On the 11th postoperative day, the patient was discharged with left upper limb muscle strength grade 0 and left lower limb muscle strength grade 2.

**TABLE 1:** The values of troponin I (ng/mL) and BNP (pg/mL) 6 days after surgery.

Postoperation	1day	2days	3days	4days	5days	6days	7days
Troponin I (ng/mL)	7.2	6.01	4.15	2.63	1.8	0.66	0.22
BNP (pg/mL)	932.78	840.08	595	622.39	636.81	740.63	550.12

## 3 Discussion and conclusion

We report a case of a patient who underwent coronary stent implantation 4 days prior due to acute myocardial infarction (AMI) and subsequently required emergency mechanical thrombectomy for acute ischemic stroke (AIS), illustrating the challenges of asynchronous cardio-cerebral infarction (CCI) management. Omar et al. first proposed the concept of CCI in 2010 through a case report describing a middle-aged male who experienced acute inferior wall right ventricular myocardial infarction and extensive cerebral infarction within 1 hour, and pointed out that there was a clear pathological and physiological association between the two rather than an accidental event (Omar et al., 2010). With the deepening understanding of the mechanism of cardiovascular and cerebrovascular comorbidities, CCI is now classified into two types: synchronous CCI, AMI and AIS occurring successively within a very short period of time, defined as AIS occurring within 12 h after AMI, or AMI occurring within 6 h after AIS; asynchronous CCI, the time window between AMI and AIS has not been unified, and literature reports range from 48 h to 3 months. The pathological mechanism of CCI involves multiple factors that interact with each other, such as neurogenic, cardiogenic, and systemic inflammatory responses. As a critical illness that simultaneously affects cardiac and brain tissues, it has a narrow treatment time window, complex clinical decision-making, and extremely poor prognosis, with a mortality rate significantly higher than that of simple AMI or AIS (Habib and Elhout, 2023; Obaid et al., 2019). Therefore, the challenges in the diagnosis and management of CCI far exceed those brought about by a single disease entity, and there is a lack of unified diagnostic criteria and anesthesia management guidelines, as well as guidance for corresponding anesthesia management. Reasonable anesthesia and intraprocedural management are important. We report a case of a patient who underwent coronary stent implantation 4 days ago due to “myocardial infarction” and is now scheduled for mechanical thrombectomy of the middle cerebral artery. Preoperative assessment of the heart and judgment of the correlation with cerebral infarction are particularly important to guide anesthesia management. Echocardiography revealed an attachment from the apex of the left ventricle, which was considered a thrombus. The key to anesthesia is to maintain effective perfusion pressure, avoid hypotension, and minimize large fluctuations in preload, afterload, and heart rate to prevent heart failure. Anesthesia aims to maintain stable hemodynamic fluctuations and effective cerebral perfusion pressure.

### 3.1 Diagnosis of cardio-cerebral infarction (CCI)

The main causes of CCI include atherosclerosis, neurogenic factors, cardiogenic embolism, systemic inflammatory responses, and certain systemic diseases. Among the causes of cardiogenic embolism, the core aspect is cardiogenic embolism after acute myocardial infarction and circulatory dysfunction, which leads to abnormal local ventricular wall movement or aneurysm formation (especially involving the apex or anterior wall), and blood flow stasis promotes thrombosis (Vaitkus and Barnathan, 1993). The hypercoagulable state activates the coagulation cascade reaction, and the detachment of the left ventricular thrombus can embolize intracranial arteries through the systemic circulation, causing acute cerebral infarction. Neuroendocrine activation also occurs during this process. After acute myocardial infarction, catecholamine release increases, exacerbating platelet aggregation and thrombosis and further increasing the risk of AIS (Boyanpally et al., 2021; Aggarwal et al., 2021; Akinseye et al., 2018). In this case, the diagnostic consideration for asynchronous CCI. First, there was a clear temporal sequence, with AIS occurring 4 days after AMI, fitting the typical window for asynchronous CCI. Second, and most critically, preoperative transthoracic echocardiography identified a left ventricular apical thrombus. This finding provides the direct pathological link, demonstrating that the cerebral infarction was a cardioembolic event originating from the recent myocardial injury. Third, common alternative causes of AIS, such as atrial fibrillation, significant carotid stenosis, or hypercoagulable states unrelated to AMI, were not present in this patient. Therefore, the AIS was not an isolated thromboembolic stroke but a direct complication of the AMI, fulfilling the core premise of CCI.

### 3.2 Selection of anesthesia method

The choice of anesthesia technique in AIS thrombectomy remains a subject of debate, with guidelines suggesting a nuanced approach. The 2023 SFAR consensus recommends that general anesthesia with intubation should be preferred in specific high-risk situations, including diminished mental alertness, agitation, vomiting (Quintard et al., 2023). Our patient presented with drowsiness, slight agitation, and a significant neurological deficit, which would traditionally favor GA. However, considering his recent AMI and compromised LV function, we were particularly concerned about the potential hemodynamic fluctuations associated with induction of and emergence from intubated GA, which could exacerbate myocardial strain or precipitate acute heart failure—a major concern in cardiogenic shock management. Therefore, we opted for a ‘non-intubated general anesthesia’ approach (deep sedation with secured spontaneous ventilation), a strategy that sits between conscious sedation and traditional GA. This decision balanced the guideline’s intent to control agitation and ensure procedural success with the cardiogenic shock principle of minimizing negative inotropy and afterload changes (Sinha et al., 2025). It allowed for immediate airway control if needed while avoiding the significant hemodynamic swings of laryngoscopy and positive pressure ventilation. This tailored approach underscores the necessity of adapting stroke anesthesia guidelines to comorbid high-risk cardiac conditions.

## 3.3 Anesthesia management strategy

In addition to routine anesthesia management, special points mainly refer to CCI management, and it is necessary to consider dual cerebral and cardiac perfusion. The core measures include: ① Reperfusion therapy: Prioritize restoring cerebral and cardiac blood flow, such as percutaneous coronary intervention (PCI) combined with intravenous thrombolysis/therapeutic aspiration. ② Anticoagulation therapy: In terms of anticoagulation, early in large-scale cerebral infarction, anticoagulation is prohibited (risk of bleeding transformation is high) (Seiffge et al., 2019; Smythe et al., 2020); Post Hoc Analysis of the ELAN Randomized Clinical Trial the findings suggest Early direct oral anticoagulant C initiation (<48 h after minor and moderate stroke, 6–7 days after major stroke) for patients after atrial fibrillation-associated ischemic stroke may be more favorable (Polymeris et al., 2025). Regarding antiplatelet therapy, AMI patients should receive dual antiplatelet therapy immediately (with a loading dose followed by a maintenance dose); for AIS patients, it should be individualized based on the reperfusion method (such as starting 24 h after thrombolysis) (Granot et al., 2007). In this case, the surgeon chose to continuously infuse tirofiban into the ICU, balancing the risks of bleeding and ischemia. Although the stroke etiology was primarily cardioembolic, tirofiban was used in this case due to the concern for potential *in-situ* thrombosis or platelet activation associated with the recent coronary stent implantation and the acute ischemic event, aiming to prevent early re-occlusion. However, evidence supporting routine use of glycoprotein IIb/IIIa inhibitors in cardioembolic stroke is limited, and its benefit may be more established in atherosclerotic stroke (Qiao et al., 2025; de Almeida Monteiro et al., 2025; RESCUE et al., 2022). Although the routine application evidence of tirofiban in pure cardiogenic embolic stroke is limited, it can reduce the 30-day in-stent thrombosis rate in patients undergoing percutaneous coronary intervention and stent implantation (Heestermans et al., 2009). Moreover, clinical studies have shown that intravenous use of tirofiban combined with intra-arterial thrombolysis and mechanical thrombectomy can be used to treat cerebral large vessel occlusion, and does not significantly increase the risk of bleeding (Jiang et al., 2025). Given that this patient has a dual pathological state of recent coronary stent implantation (requiring intensified antiplatelet therapy) and acute large vessel occlusive stroke (requiring thrombectomy treatment), the use of tirofiban aims to balance the risks of ischemia and bleeding, especially to prevent early re-occlusion.③ Blood pressure management: Hypertension can exacerbate myocardial ischemia and heart failure risk, and hypotension can lead to insufficient cerebral perfusion and expand the infarction core. Therefore, the goal of intraprocedural blood pressure management is to maintain MAP at baseline ±20%, heart rate at baseline ±20%, and when necessary, combined transesophageal echocardiography/transthoracic echocardiography (TEE/TTE) monitoring of cardiac function and maintenance of coronary perfusion with restrictive fluid administration and prophylactic vasoconstrictive drugs (such as norepinephrine). In terms of the anesthesia plan, we chose non-invasive general anesthesia with sufentanil combined with remifentanil. This decision was based on the pharmacological properties of the two drugs: sufentanil, as a potent analgesic, can provide a stable analgesic base with a very small dose and has relatively mild effects on the circulation; remifentanil, as an ultra-short-acting opioid, does not rely on liver and kidney functions for metabolism and can adjust the dosage to control the depth of sedation in real time, thereby achieving rapid and predictable hemodynamic management. For this patient with severely reduced left ventricular systolic function (EF <40%), maintaining the stability of heart rate, preload, and afterload is crucial to avoid increasing myocardial oxygen consumption or triggering acute heart failure. This drug combination helps to provide adequate sedation and analgesia while minimizing perioperative hemodynamic fluctuations, which is in line with the management principles for such vulnerable cardiac patients. Other intraprocedural anesthesia points: Monitoring of anesthesia depth: gradual titration of administration to avoid overly deep inhibition of circulation; protection of fragile myocardium: avoiding excessive fluid infusion; and preferring target-oriented fluid therapy combined with vasoactive drugs.

## 3.4 Postoperative outcome and complications

Patients with CCI have significantly worse prognosis than those with simple AMI or AIS, and the main complications include acute kidney injury, hemorrhagic transformation, and gastrointestinal bleeding (with significantly increased incidence) (Ho et al., 2024; Obaid et al., 2019; Alqahtani et al., 2017). The patient was transferred to the ICU after surgery, and on the third day, when the condition stabilized, it was transferred to the general ward.

In conclusion, CCI is a complex clinical syndrome that requires close collaboration among neurology, anesthesiology, cardiology, and intensive care medicine departments. Anesthesiologists must understand the pathological damage of the cardiovascular system, the corresponding pathophysiological changes, and determine hemodynamic goals. Preoperative echocardiography is important for monitoring cardiac function. Appropriate anesthesia methods, intraprocedural blood pressure management, and precise regulation of circulation during the operation should be used to reduce the secondary impact of anesthesia on the fragile heart and brain. It is important to acknowledge the limitations of this report. As a single case study, our experience cannot establish general guidelines but rather illustrates a management strategy that was successful in this specific, high-risk context. The decisions regarding anesthesia technique and antiplatelet therapy were tailored to this individual’s unique profile of recent stent implantation, ventricular thrombus, and severe cardiac dysfunction.

Nevertheless, this case highlights that a multidisciplinary, physiology-guided approach is crucial. The anesthesia management described here may serve as a useful reference point for teams faced with similar complex decisions in the management of asynchronous CCI.

## Data Availability

The original contributions presented in the study are included in the article/supplementary material, further inquiries can be directed to the corresponding authors.
